# Meaning-Change Through the Mistaken Mirror: On the Indeterminacy of “Wundt” and “Piaget” in Translation

**DOI:** 10.1177/10892680211017521

**Published:** 2022-01-04

**Authors:** Jeremy Trevelyan Burman

**Affiliations:** 1 University of Groningen, Groningen, The Netherlands

**Keywords:** Piaget, translation, meaning-change, indigenization, critical digital history of psychology, integrated history and philosophy of science (iHPS)

## Abstract

What does *a name* mean in translation? Quine argued, famously, that the meaning of *gavagai* is indeterminate until you learn the language that uses that word to refer to its object. The case is similar with scientific texts, especially if they are older; historical. Because the meanings of terms can drift over time, so too can the meanings that inform experiments and theory. As can a life’s *body of work* and its contributions. Surely, these are also the meanings of a name; shortcuts to descriptions of the author who produced them, or of their thought (or maybe their collaborations). We are then led to wonder whether the names *of scientists* may also mean different things in different languages. Or even in the same language. This problem is examined here by leveraging the insights of historians of psychology who found that the meaning of “Wundt” changed in translation: his experimentalism was retained, and his *Völkerpsychologie* lost, so that *what Wundt meant* was altered even as his work—and his name—informed the disciplining of Modern Psychology as an experimental science. Those insights are then turned here into a general argument, regarding meaning-change in translation, but using a quantitative examination of the translations of Piaget’s books from French into English and German. It is therefore Piaget who has the focus here, evidentially, but the goal is broader: understanding and theorizing “the mistaken mirror” that reflects only what you can think to see (with implications for replication and institutional memory).

What does *a name* mean in translation? Quine (1960) argued, famously, that the meaning of *gavagai* is indeterminate until you learn the language that uses that word to refer to its object: whether this is meant to indicate a rabbit, rabbitiness, or something else, is unclear. For the translation of scientific texts, this implies that we cannot really know—without sophisticated language skills—whether English science is *identical with* French science or with German science. Unless, that is, we can leverage the special meaning of *science* and replicate the original source’s findings to equivalent effect. Replication thus becomes a practical alternative for bilingualism (perhaps also for clear writing) and it accommodates the tacit by replacing apparently irrelevant references to unknown names and sources then reconstructing their scientific claims within the new framework that seeks to reproduce them. *Science* works, in other words, because the *meanings* of our names for objects are *secondary*. Or so we have been led to believe.

So let us pursue this line, lest we be misled by the current focus on results-replication over meaning-preservation, following what Quine (1960) relatedly called the “inscrutability” of reference (pp. 48, 69–72). Thus, for example, we might render this as the problem of *understanding* scientific *texts*, prior to the replication (perhaps only through “virtual witnessing”)^1^ of what they describe. This is then a challenge not solely because it is unclear what texts mean in translation, but also just if they are older; historical. Because *referring-terms* can drift over time. They can even reverse in meaning, as in the case of “genetic” (which for Baldwin, Hall, and Piaget meant *developmental* in the sense of “genesis” or “generation” and only later came to mean *evolutionary* in the sense of “pertaining to genes”). As a result, the fundamental categories governing what it is that should be replicated can change too: the same word can be used in different contexts without clarity regarding the identity of its meanings.

This has a parallel in the classic problem of “incommensurability,” in which comparisons can be made but meanings are lost in the process (following Kuhn, 1962/2012, 1983/2000, 1999). In such cases, relying on replication as an alternative to understanding is therefore to invite failure.

Of course, we can make the problem worse. We might ask about the theories or meta-theories, or the procedures or technologies, that are embedded in and inform experiments tacitly (even obviously) for one group but not for another. Or we might ask about a life’s *body of work*, during which almost everything—experiments, theory, meta-theory, procedures, tools, and techniques—may have changed. Surely these also afford the meanings of a scientific name that need to be interpreted, if only as *cognitive short-cuts* to fuller descriptions of the author who produced them or of the thinking that informed the reported view of their objects (maybe also of their collaborations, suppliers, patrons, and social networks). We are thus led to wonder whether the *names* that index the *contributions of scientists* may mean different things at different times in different languages, or even at different times in the same language. And so too must we ask about the special meaning of science, in which—following the popularity of Thomas Kuhn’s (1962/2012) *Structure of Scientific Revolutions*—replications can only be reasonably expected to work within “paradigms” (or, more aptly given what follows, “thought collectives” in Fleck’s [1935/1979] looser sense).^2^

This problem is examined here by leveraging the insights of historians of psychology who found that the meaning of “Wundt” changed in translation: his experimentalism was retained, even as his *Völkerpsychologie* was lost, and so *what he meant* was altered even as replications of his work—and the continued use of his name—informed the disciplining of Modern Psychology as an experimental science. Those historical facts and insights are then turned into a general argument, regarding meaning-change in translation, but using a quantitative examination of the different translations of Jean Piaget’s books from French into English and German. It is therefore “Piaget” who has the primary focus here, evidentially, but the goal is broader: understanding and theorizing “the mistaken mirror” that reflects only what you can think to see (as Leahey, 1981, put it of Wundt’s replication by Titchener).^3^

## The Wundt Argument

To begin with another classic example, let us consider Frege’s (1892/1980) reflection on the difference between “the morning star” and “the evening star.” The *reference* is the same in both cases because both descriptions refer to the planet Venus as it hangs in the sky overhead. But the *sense* is not the same. This is because—to update Frege’s references in Greek to the now-more familiar Latin of the Judeo-Christian tradition—“morning star” can refer to Lucifer, which in Latin literally means “the light bringer” but also has additional connotations^4^ via that tradition which the Greek “Phosphoros” does not.

I propose much the same thing for the meaning of the name “Wundt” in the history of psychology, although without casting him reverentially^5^ as a fallen angel. And further, I propose that this is the result of translating and reconstructing the contributions of the designated man—the German professor of philosophy named Wilhelm (1832–1920)—in a new context where he had no physical presence. In the process, his “foreign” interests were neglected (Anderson, 1975; Blumenthal, 1975, 1977; Danziger, 1980). And the resulting reconceptualization then stood-in for the original source. Although the *reference* remained the same, the *sense* was different.

To put this more plainly, “Wundt” was *Americanized* by his American followers (see Leahey, 1981; Rieber, 1980; Tweney & Yachanin, 1980). *Their* critics then dismissed *him* as well, and it’s this—not Watson—that gave us Behaviorism (see Burnham, 1968; O’Donnell, 1985). The resulting *frame of reference* in turn also gave us what we call the Cognitive Revolution (see Baars, 1986; Gardner, 1985). Even though what happened at that time did not meet the technical definition of a “revolution” in science (Greenwood, 1999; Leahey, 1992).

In other words, the consequence of this Americanization is not about the *object* “Wundt.” The man himself did not actually move to the United States.^6^ What is at issue, instead, is the *meaning* that refers to *the name* and thus also the *implications* of that name: the *sense* that the name has, in translation, separate from *the man* that it refers to.

For an Americanized Wundt to be “the founder” of American Psychology constrained what the resulting discipline could be defined to be. The discipline was therefore directed down one path over another: toward “experimental” research, rather than an “applied” approach (O’Donnell, 1979). Thus, by that reference, the meaning of psychology was also made scientific; “objective” (Green, 2010).

Textbooks in psychology have traditionally been written to tell this story in greater detail, albeit while presenting what I am referring to as *meanings* as *facts-in-themselves*. The alteration to that standard story that I am proposing is therefore this: the discipline was refashioned, during its importation, to reflect the relevant values that Wundt’s *name* was understood to *represent*,^7^ but without as much regard for what *the man* himself had actually said (via Titchener but also especially after their further popularization by Boring, 1929, 1950). It was therefore also by this means that we arrived at the present perspective: Wundt’s metaphysics were repudiated, and yet his name nevertheless became synonymous with psychological science (Danziger, 1979, 1990).

To turn momentarily reflexive, in considering the discipline’s origin story, the clustering of my references in the preceding paragraphs—primarily in the late 1970s and early 1980s, with shorter articles slightly earlier and longer books slightly later—is also itself meaningful. In short: after decades of having been influenced by Titchener and Boring, the realization that meaning-loss had occurred followed as a result of the interest generated by the centennial of the founding of Wundt’s lab at Leipzig in 1879. This then became a matter for historical investigation, leading up to the celebrations set for 1979, and psychologists with historical interests learned of the omissions in the importation. Especially of the *Völkerpsychologie* (see Danziger, 1983; Farr, 1983). This didn’t close the book on those historical investigations, however, because *those omissions* continue to be of compelling research interest (e.g., Greenwood, 2003; Kusch, 2019; Wong, 2009).

My argument follows simply and incrementally from there: what American psychologists understood of Wundt was a function partially of the prevailing interests, rather than solely of what the primary sources indicated. Certain ideas were imported, and others were not. Furthermore, I propose that these interests themselves caused meaning-change in what was imported as previously relevant aspects of Wundt’s approach were dismissed as irrelevant in the new context. As a result, in other words, the impact was of an imported *sense*, not of the original *reference*. And I propose that something similar happened with the Swiss natural historian and psychological philosopher Jean Piaget (1896–1980). Developmental Psychology and Educational Theory were then pushed to develop in directions that were more consistent with the receiving audience’s interests than with what was expressed in the original texts.

Briefly put: the majority of Piaget’s translated books were imported into American Psychology during and after what has variously been called the “resurgence,” “revival,” and “rediscovery” of Piaget in the 1950s and 1960s (see Müller et al., 2013, p. 52). Their meanings were therefore reinterpreted through the Cognitive Revolution. And that in turn explains the lopsidedness of Piaget’s legacy (Bond & Tryphon, 2007). It also provides a plausible explanation for the subsequent interest in the existence of a “new” theory: in the period leading up to the centenary of Piaget’s birth, in 1996, insiders became aware that the contemporary view of Piaget’s works was skewed. They then went looking for what had been missed, in much the same way as the Wundtians had, and found it (e.g., Beilin, 1989/1992, 1992a, 1992b; Brown, 1988; Davidson, 1988; Dean & Youniss, 1991; Vidal, 1994).^8^ In other words, Piaget was *also* “Americanized” (see also Rowland, 1968; Voyat, 1977).

My goal here, however, is not to summarize that second history of invisibility and neglect.^9^ Instead, I want to offer a general version of what we might call “The Wundt Argument” (psychology changed as it was imported) that can be understood to apply to other authors whose works were also translated and popularized. To do this, I have replicated Josef Brožek’s (1980a, 1980b)^10^ citation analysis of Wundt’s impact using citations to translations of books by Piaget. In addition, because I also want to use these data to make an argument about the *form* of science, rather than its *content* (which is framed by the form),^11^ I have gone a step further: my purpose here is not simply to report historical facts, but also to use them as evidence of the larger set of concerns that must be investigated whenever scientific meanings are moved (including during replication). I have therefore provided several graphs to help support the “virtual witnessing” of my argument, although I have done so with the full knowledge that they are so visually complex as to be nearly uninterpretable without the accompanying text; rather than being left to speak for themselves, they are read-in and used as evidence to make my larger point.

## Generalizing From Wundt to Piaget

Brožek (1980a) situated his study of Wundt’s impact as part of the Leipzig Laboratory’s centennial celebrations, but he also explicitly distanced himself from its reverential atmosphere. As he explained, “my aim was (and is) to contribute new factual information, not to join the chorus” (p. 103). To gather his data, he then examined the first 90 volumes of the *American Journal of Psychology* for citations. And he did so by hand: none of the automated tools that now exist could be appealed to and applied.^12^

The details of what he found are not at issue. What is interesting for our purposes are the rankings he produced of Wundt’s books. This, for Brožek, was a way to confirm that the well-known *Grundzüge der physiologischen Psychologie* (translated by Titchener as *Principles of Physiological Psychology*) was actually Wundt’s “bestseller” (p. 105). Indeed, Brožek equates its popularity—with 61% of all citations in his sample—with its “relative importance” (p. 106). This, then, is my focus: not the raw counts, but the relative rankings.

We know from subsequent historical work that Brožek’s (1980a) aside about the *Völkerpsychologie* having “fared poorly” (p. 106) would soon be given a new interpretation: Titchener left things out on purpose (Leahey, 1981). Seen from that perspective, Brožek’s quantitative data can then also be understood as providing evidence not of each book’s “importance,” but of their “reception.” (Their *sense*.) Therefore, in retrospect, I would also reinterpret Brožek’s data as being about *each book’s perceived-importance relative to the extant interests* (i.e. reflecting especially the influence of Boring, 1929, 1950). And that is what I have done for Piaget: the citations provide evidence of what the audience of importers found most interesting or useful (or relevant).

To enable such a comparison, I collected the citation counts provided by Google Scholar^13^ for all of Piaget’s books in French and for their English and German translations when those existed.^14^ The resulting perspective is a bit skewed toward the present, of course, because Google has access only to those materials that have been digitized. (And also because the disciplinary “style” for citations has changed over the period of study; see Sigal & Pettit, 2012.) But this at least should be skewed consistently: Google has no particular interest in any one aspect of Piaget’s output over another, except perhaps for a preference for recent citations, so its data should be more than adequate for the purposes of my demonstration. Indeed, the numbers themselves are not what matters, for my purposes here, so long as they skew *consistently* (cf. Burman, 2018b, on the changing consistency of PsycINFO’s meta-data).

## A Formalized Comparative History

The great benefit of this approach is the very large “n.” Indeed, at the time of my first data collection, I found 104,814 citations distributed across all three languages. This then affords an easily falsifiable kind of null hypothesis: if Piaget’s texts were *not* imported in the same way that Wundt’s have been shown to be by other means, and instead were brought over in ways that reflect their *true* importance, then their impact—as assessed by citation counts—should be *the same* in French, English, and German. (Or rather, the number of citations received by each book would be the same relative to the total number of citations made in each language; the *ratios* should be the same.)

**Figure 1.**
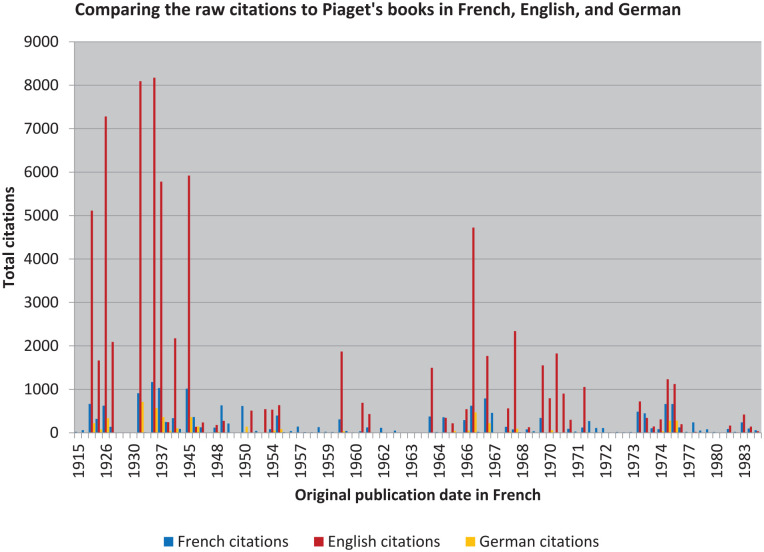
Piaget’s raw citation data, in three languages, with no manipulation. *Note.* Dates are according to original source publication. There is one exclusion: *The Psychology of Intelligence* (Piaget, 1947/1950), for reasons explained below.

**Figure 2.**
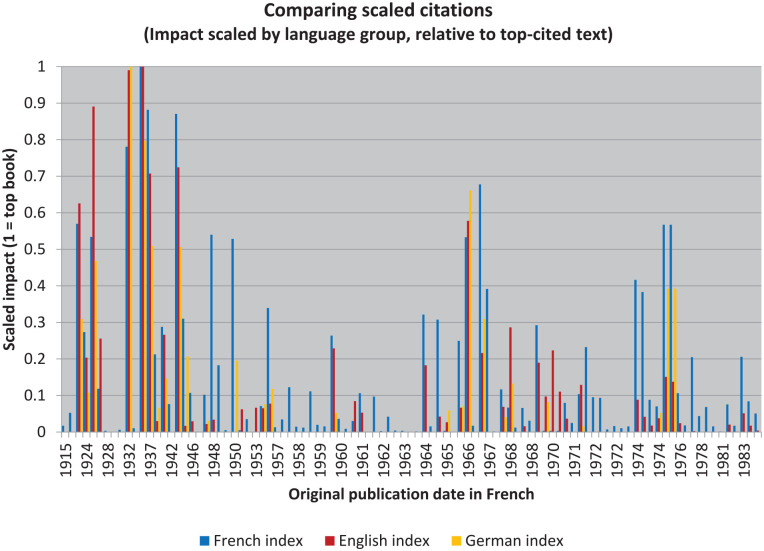
When each language is treated as a single group, the raw counts can be compared between them as ratios.

Needless to say, however, that is not the case (see Figures 1 and 2). Nor am I interested in *true* importance: my focus in what follows is on observing and explaining the relative differences in *reception*.^15^ That is then also the source of my key investigative insight: to use translations of the same texts as *variora* received in different contexts at different times. We can therefore take advantage of the citation numbers as proxies not so much for these texts’ meanings, directly, as for the consequences of each audience’s in-context interpretations of those texts’ relative importance: the more highly cited is a text in a collection, the greater is its *perceived significance* for that language group. Then, because different translations of the same text can be considered formally identical with themselves, or at least *equivalent* (following Quine), differences in relative position considered against the backdrop of a receiving (hidden) value-hierarchy then suggest differences in received meaning that can be investigated using more traditional historical methods (e.g., Burman, 2021).

### The Raw Data, and the Cooked

To replicate Brožek’s (1980a) study, the first step is to present a simple ranking using the raw data from Google Scholar. This, though, already highlights a problem that plagues digital histories: at the time when these data were first collected, in early 2013, there were many thousands of citations reported for the English translation of *The Psychology of Intelligence* and very few for the French original (Piaget, 1947/1950). But when a second replicating round of data collection was performed in early 2015, the situation was reversed: thousands of citations for the French, and very few for the English.^16^ In a third examination of the data, undertaken in late 2016, the underlying issue seemed to have been resolved and remains so at the time of this writing. However, the inconsistency itself seems fatal for those who trust these numbers uncritically. Of course, problems like this are actually fairly common in the digital humanities: getting the data is just the beginning.

Corrections to “raw” data are almost always required to produce trustworthy results.^17^ Indeed, this need is so pervasive that one commentator pointed out that “raw data is an oxymoron” (Gitelman, 2013). In all such studies, in other words, the data are always at least lightly “cooked.” (Brožek’s, 1980a, study is full of selections and exclusions.) Still, it is worth noting that no other book seems similarly affected. I have therefore simply accepted the common view that Piaget’s *The Psychology of Intelligence* was akin to Wundt’s *Grundzüge* in its impact, and moved on to a deeper analysis using the data that remained consistent across all three examinations. A brief word, then, about what it is that we are excluding.

As Piaget’s books go, *The Psychology of Intelligence* is relatively simple. It was developed originally as lectures for a course that he taught at the College de France in Occupied Paris, in 1942, as part of his resistance to World War II: “at an hour when university men felt the need to show their solidarity in the face of violence and their fidelity to permanent values” (Piaget, 1947/1950, p. vi). Publication and translation were delayed until after the war was over.

This was then the first of Piaget’s books to appear in English since the translation of *The Moral Judgment of the Child* almost 20 years before. (The British edition was published as Piaget, 1932/1932, although the American edition was delayed an additional 30 years.) Given this, it is no wonder that *The Psychology of Intelligence* was so influential: it marked Piaget’s return to English psychology. Indeed, he received an honorary doctorate from the University of Chicago for it in 1953 (see Burman, 2021).

In German, however, the situation was somewhat different. Translated almost immediately, and published in 1948, *Psychologie der Intelligenz* was the first-ever German-language edition of one of Piaget’s books and the first of nearly three dozen translations. It can therefore be understood to occupy the special position of having provided the first impression of Piaget for German audiences. Simply put: every book published afterward would have been read through its influence, despite the preexistence in French and English of earlier books (Because of what we might call the Levi Effect: a version-in-translation, with alterations and omissions, of “the Matthew Effect” [see Merton, 1968]).

Comparing the reception of the three editions, the relatively lower number of citations received by the book’s English translation (where it would rank third on our Brožekian bestseller list, instead of first) can perhaps be attributed to the popularity of John Flavell’s (1963) introductory textbook: *The Developmental Psychology of Jean Piaget*. This is widely recognized as having been the first major work dedicated solely to making Piaget accessible to English-speaking audiences. The two books also cover very similar territory. Indeed, commentators on the draft of 1957 dismissed it as having been skewed in its perspective by that book’s recent translation (see Müller et al., 2013, p. 52). And this in turn provides a possible explanation for the English edition’s slightly lower ranking on our initial list: Piaget and Flavell competed for citations in English, but—because no translation of Flavell was made—they did not compete in French or German. (When Flavell’s book’s citations are added to Piaget’s, the combination then also ranks first in English.)

From this perspective, what “Piaget” means for contemporary audiences is *old*: his ideas are WWII-era, despite his having continued to stay active in research until his death in 1980. At the same time, Piaget’s view is also clearly *foreign*. Rather than treating intelligence in psychometric terms, he considered it to be the consequence of a developing sequence of “operational” structures and groupings (see Burman, 2021).

That is not archaic, from an American perspective; it is *odd*. For an Historian interested in differences, though, it is ideal: when you look, you don’t see what you expect. Still, as an introduction to the ideas that informed Piaget’s “standard theory” of stages, one could do much worse than *The Psychology of Intelligence*. Now, though, we want to see beyond its influence. Excluding it from our analyses is therefore not only necessary for practical reasons, related to a distrust of the underlying data, but it is also very useful: we do not want to be blinded by its influence, and—if it is indeed the equivalent of Wundt’s *Grundzüge*—the omission will mean that our illustrations will not be compressed by its relatively large “n” (consistent with Zipf’s Law; cf. Benjafield, 2016).

### Basic Figures

To derive the remainder of our first bestseller list, we can report some basic figures. Thus, for example, the means and standard deviations for citations to translations of Piaget’s other books having editions in French, English, and German were 186.2 (261.1), 1,461.4 (2,114.5), and 172.6 (182) respectively.

If this difference generalizes to other authors, it immediately suggests a reason for why funding agencies are usually keen to have their grantees publish in English: Piaget’s impact in translation is nearly an order of magnitude greater than it was in French (or in German). But the differences also provide us with a simple way of assessing the impact of books across different languages: it is clear that each language group needs to be treated separately before the *variora* can be compared as equivalents. Thus, those books that received more citations than two standard deviations (**) above the mean in each group are listed in Table 1, along with their translated titles, as a convenient shortcut for limiting our lists to “the most significant” books.

**Table 1.** Piaget’s “bestsellers” by citation count (total citations in that language), not including *The Psychology of Intelligence*.

French	English	German
*La naissance de l’intelligence chez l’enfant*, 1936 (1,167)*** The origins of intelligence in children, trans 1952	*The origins of intelligence in children*, 1936/1952 (8,174)***	*Das moralische Urteil beim Kinde*, trans 1932/1954 (711)** [The moral judgment of the child]
*La construction du réel chez l’enfant*, 1937 (1,029)*** The construction of reality in the child, trans 1954	*The moral judgment of the child*, 1932/1932 (8,093)***	*Das Erwachen der Intelligenz beim Kinde*, 1936/1969 (567)** [The origins of intelligence in children]
*La formation du symbole chez l’enfant*, 1945 (1,016)*** Play, dreams and imitation in childhood, trans 1962	*The child’s conception of the world*, 1926/1929 (7,280)**	
*Le jugement moral chez l’enfant*, 1932 (911)** The moral judgment of the child, trans 1932	*Play, dreams and imitation in childhood*, 1945/1962 (5,921)**	
*Biologie et connaissance*, 1967 (791)** Biology and knowledge, trans 1971	*The construction of reality in the child*, 1937/1954 (5,781)**	

Despite the obvious differences in “n,” the consistency at the top of each of the language lists is remarkable: *The Origins of Intelligence in Children* (Piaget, 1936/1952) and *The Moral Judgment of the Child* (Piaget, 1932/1932) appear in all three, and—if one is interested solely in identifying the Great Books by Psychology’s Great Men—they are clearly key companion texts to read alongside *The Psychology of Intelligence*. But they are also very early texts. In fact, with only one exception related to the French reception of the book translated as *Biology and Knowledge* (Piaget, 1967/1971), all of the “bestsellers” were originally published prior to the end of WWII.

### Citation Density

A simple next step is to calculate a slightly different kind of bestseller list by accounting for the number of years since publication, and thereby introducing a control for the Matthew Effect’s tendency to accumulate a greater number of accolades for texts made available and recognized earlier. This then also allows us to get to a more temporally sensitive version of what Brožek (1980a) referred to as “the relative ‘weight’ of . . . individual publications” (p. 103).

Citation density indexes the impact per year in which it was possible to cite a text. This can be calculated easily by dividing the total citation count by the number of years since publication. That then provides a time-controlled metric that enables the comparison of works produced at different times across a long life span, although it is especially useful when comparing translations made of the same text that were themselves published at different times (thereby accruing different citation periods and even different orders of publication). It also provides a better assessment of “momentary bestsellerness” than does the raw citation count: a more exciting recent text will have a greater number of citations per year, even though it may also have fewer total citations as a result of its recency.

We can again use means and standard deviations to quickly narrow in on “the most significant” books in the group. Thus, to summarize: Piaget’s original French works were cited an average of 3.5 times per year since publication (with a standard deviation of 4.6 citations per year), English translations 28 times per year (33.2), and German 4 times per year (4.1). Focusing on the works that received the greatest number of citations per year, again using the arbitrary cut-off of two standard deviations (**), then gives the results in Table 2.

**Table 2.** Piaget’s “bestsellers” by citation density (cites per year since publication), not including *The Psychology of Intelligence*.

French	English	German
*L’équilibration des structures cognitives*, 1975 (17.9)*** Equilibration of cognitive structures, trans 1978 and 1985	*The origins of intelligence in children*, 1936/1952 (136.2)***	*Die Psychologie des Kindes*, 1966/1977 (13.4)** [The psychology of the child]
*Biologie et connaissance*, 1967 (17.6)*** Biology and knowledge, trans 1971	*The psychology of the child*, 1966/1969 (109.8)**	*Das Erwachen der Intelligenz beim Kinde*, 1936/1969 (13.2)** [The origins of intelligence in children]
*La naissance de l’intelligence chez l’enfant*, 1936 (15.4)** The origins of intelligence in children, trans 1952	*The construction of reality in the child*, 1937/1954 (99.7)**	*Das moralische Urteil beim Kinde*, 1932/1954 (12.3)** [The moral judgment of the child]
*La psychologie de l’enfant*, 1966 (13.5)** The psychology of the child, trans 1969		
*La prise de conscience*, 1974 (12.8)** The grasp of consciousness, trans 1976		

These results are interesting in a different way: the French originals are both more numerous and more diverse than the English or German translations. (This is to be expected from the point of view of theories of cultural evolution: the oldest source typically shows the greatest diversity.) And although the highest “density” books from the English and German lists are contained within the French list, the French list also includes more recent books.

There are, as before, some details here that need to be examined. Indeed, the French #1, *L’équilibration des structures cognitives* (known in English as *The equilibration of cognitive structures*, Piaget, 1975/1985), requires some explicit discussion.

This book is the first theoretical statement, on the psychological side, of “Piaget’s new theory” (see especially Beilin, 1992b). It reviews and integrates the first three volumes of experiments from that period (viz. Piaget, 1974/1976, 1974/1978, 1974/1980; see Ducret, 2000). It also replaced the model developed during the first period of research in genetic epistemology (viz. Apostel et al., 1957; see Burman, 2021). Furthermore, it represented Piaget’s return^18^ to theorizing the primary constructive mechanism required of his system: “*équilibration majorante*,” which—lacking explicit access to the updated logic of just a few years prior (see Burman, 2016)—previous translators had rendered unhelpfully as “optimizing equilibration.”

Perhaps unsurprisingly, given my focus here, this translation is insufficient to convey the intended meaning. Among other things, it misses the transcending step implied by the level-change that was made more explicit in Piaget’s other works from the era.^19^ Yet, even with those sources in hand, this book would still have been very difficult to explain: as a summary, it assumes a huge amount of material that is not discussed explicitly and remains only alluded to. This lack then had real consequences in moving its meaning across contexts.

In English, this book has the dubious distinction of having received two translations. This was necessary because, to put it plainly, the first attempt was “seriously defective” (Smith, 2009, p. 28). What’s worse, though, is that—at the three moments when these citation data were examined—the earlier translation had actually received more citations than the higher-quality replacement. (This finally reversed during the final preparation of this article.) The result is that, until recently, the majority of readers in English had not read even an approximation of the text read by French audiences. In other words, despite my methodological idealism in considering *variora*, those two versions cannot even be considered equivalent.

Given the book’s position on our second bestseller list, this is itself quite significant. But it is not the only *variorum* with problems. For example: one of Piaget’s best-known translators, Eleanor Duckworth, observed that *Origins of Intelligence—*which sits at #1 on both of our English bestseller lists—also needs to be replaced (noted in the English translation of Ducret & Schachner, 2011, pp. 1n–2n). Doubtless there are many others too: the translators of *Equilibration* and *Origins* were responsible for several volumes, including the bestselling *The Construction of Reality in the Child* (Piaget, 1937/1954). It therefore seems likely that many of Piaget’s books contain substantial divergences from the originals. These, however, are not my focus here (see Jurczak, 1997; Smith, 1981, 2009).

### Comparing Across Language Groups

Listing top-ranked publications is easy so long as one has access to the data. But we want to compare *all* of Piaget’s books, to the extent that they exist in multiple languages, in a systematic and direct way. As a result, we must now begin to move beyond what Brožek (1980a) attempted, although—because the technology is quite different today—we can also do so simply by taking advantage of the available computational tools.

In addition: the recognition that all such data are “cooked,” to one extent or another, enables us to play with the numbers in ways that are more revealing than would be the raw citations on which most such studies focus. Yet the resulting manipulations are mathematical (arithmetical), rather than statistical. (I’ve used statistics only descriptively, and only where this adds clarity.) They are also as simple as can be managed: although more complex manipulations might reveal more, their value for our understanding would decline in proportion to their complexity. As, indeed, we can see in the resulting pictures.

The first manipulation, then, is a kind of scaling. This is necessary if we are to compare across language groups. And it requires only that the citation count for each book be considered as a ratio relative to the most highly cited book in that group.^20^ This allows for the relative impacts in each language to be compared without concern for the external causes of differences in citation patterns (e.g., the number of people working in each language group or their generosity in citing).

Presenting these results graphically is relatively simple. The tallest bar of each color represents the highest-impact text in that language group. But the results are indeed powerful: they afford the first real test of our null hypothesis. Simply put: had the impacts been the same in each language, as one might expect if relative impact could be used as a stand-in to describe the books themselves, then the bars in Figure 2 would be flat three-across for each book. That is clearly not the case: the different language-groups received the books in different ways.

To make the point conclusively, one might be tempted to add error bars and asterisks showing significant differences (or to represent the degree of certainty one has that Google’s sample is representative of the population of published citations). But these graphics are already overly complex. And it is not necessary to make them busier still: these digital techniques are intended as an investigative aid, not as a replacement for careful or deliberate thought (see Burman, 2018a, 2020c). An example will therefore suffice to make the value clear.

Looking to the first experimental books of Piaget’s new theory period, or the Piagetian equivalent of Wundt’s first *Völkerpsychologie* volumes—*The Grasp of Consciousness* (Piaget, 1974/1976) and *Success and Understanding* (Piaget, 1974/1978)—we see that they are widely cited in French, and yet hardly known at all in English. But if texts were received according to their significance, following the judgment of those who could reasonably be said to have understood them in their original vernacular rather than according to whatever meaning that they have in translation (i.e. by *reference* rather than by *sense*), then the scaled impact ought to be very similar.

**Figure 3.**
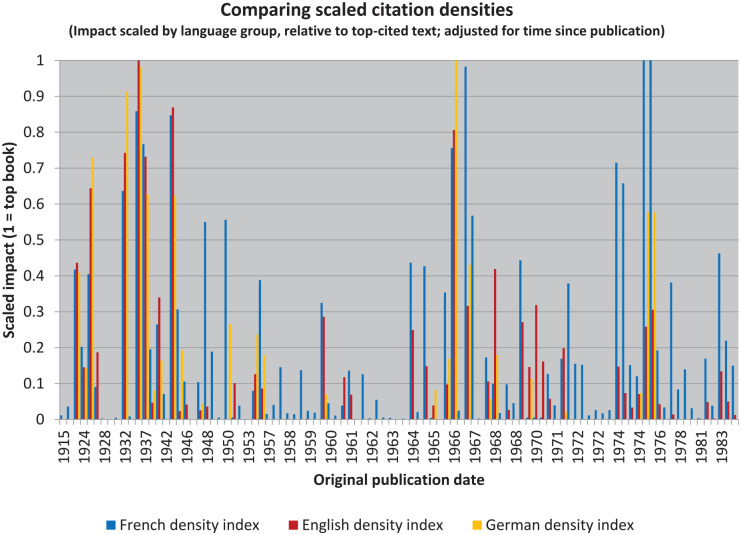
Examining the texts in their separate contexts shows they had different impacts.

In addition to rescaling and comparing the raw citation data, we can also take the further step of rescaling and comparing the calculated citation densities (Figure 3). The effect is then to normalize the impact relative to their different publication dates in each language.

Something to note quickly: because there were two versions published in English of *L’équilibration des structures cognitives (The Equilibration of Cognitive Structures)*, it appears twice in the graph. This is the double-bar located at 1975. Even despite this potential confusion, however, it is clear that the French texts are much more highly represented more recently: the graph is obviously “bluer” on the right. Indeed, with only a few exceptions, the most densely cited English and German texts are still mostly older: pre-WWII. To say more, though, we need to continue to dig into the data.

### Controlling Translation Impact With Source Impact

To assess meaning-change is to make a comparison between source and target. For Wundt, this has mostly been done qualitatively: historians realized that the target group (American psychologists and textbook authors) had gaps in their understanding relative to the sources (German psychological and philosophical texts). Brožek (1980a) then showed this quantitatively by making lists. Certain themes were clearly more popular than others, and others were simply missing (esp. the *Völkerpsychologie*). My replications and extensions with Piaget have been more complex, but the resulting comparisons have so far been very similar. We can just go further as a result of having access to more powerful tools.

To build my argument, I have added a new layer of complexity at each step. Until this point, however, they have each been simple. Now, though, we have to do something complicated. Briefly put: to make a quantitative comparison between *the contexts* that afford the differences in *sense*, rather than between texts that reflect the same *reference*, requires controlling for the impact of the source texts in the source language while at the same time presenting the impact of the translation in the target language. This cannot be done using the raw citations. Instead, we need to use the scaled language-controlled data. In what follows, we are therefore leveraging everything done to this point.

**Figure 4.**
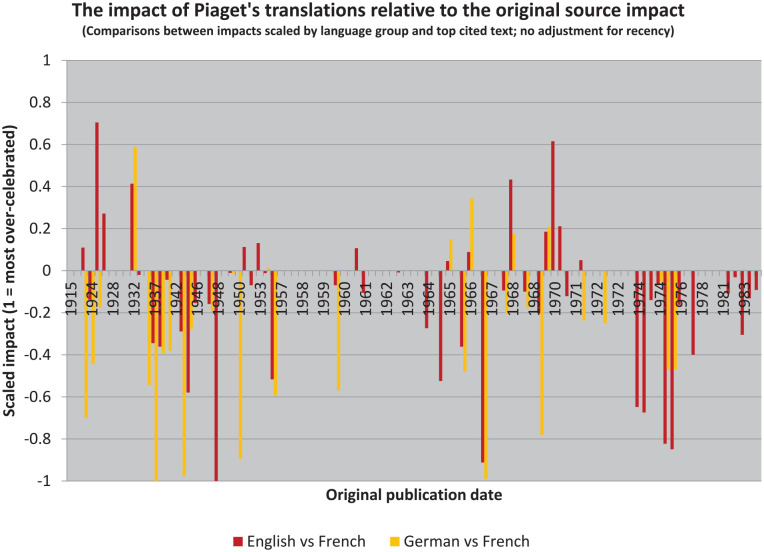
The translations have generally been received in ways that had less impact than the original sources, but not universally. A small number of Piaget’s books have had much greater impact in translation than would be expected given their reception in the source language.

The resulting illustration (Figure 4) is a kind of scaled “over-under.”^21^ Where values are negative, sources are cited more highly than translations. That the overall trend is more negative than positive, for both target languages, then suggests that the impact of Piaget’s writings has in the aggregate been reduced in translation (even though his English translations have been cited more often).

These results also provide the means to construct two new lists, which will be useful in simplifying the illustration. But these lists are also quite different from the earlier ones. Rather than identifying “bestsellers,” as Brožek (1980a) did, we can instead identify those books which have been “celebrated” in translation and those which have been “neglected” (cf. Burman, 2015).

By this I mean that their reception in translation is at odds with their reception in the original French. Following contemporary historians’ discomfort with “celebratory” narratives, however, I will instead refer to the reception of these texts “over-rated” and “under-rated” (i.e. relative to the impact of the source text in the original language). Thus, we see that the graph is generally “yellower” on the left and “redder” on the right, but also that these colors are overrepresented in both cases below the zero-line (and that they extend further below it than above it).

Inclusion on the “over-rated” list suggests that rather too much fuss has been made of these books in translation, relative to their original impact in French (see Table 3). And with the exception of *Genetic Epistemology*—a short collection of four lectures that was intended to summarize dozens of volumes, which was also delivered directly to an American audience (Piaget, 1970/1971)—the texts are known to us from earlier analyses: both *The Child’s Conception of the World* (Piaget, 1926/1929) and *Das moralische Urteil beim Kinde* (*Moral Judgment*; Piaget, 1932/1932) are on our first list of bestsellers (Table 1), and the latter is on our second list too (Table 2).

**Table 3.** Piaget’s most “over-rated” texts (by calculated score), presented in limited form using standard deviations as a shortcut for determining significance.

English vs. French	German vs. French
*The child’s conception of the world*, 1926/1929 (0.70)**	*Das moralische Urteil beim Kinde*, 1932/1954 (0.59)** [The moral judgment of the child]
*Genetic epistemology*, 1970/1971 (0.62)**	

By contrast, inclusion on the “under-rated” list suggests that rather less has been made in translation of those texts than perhaps was warranted given the way in which they were received in French (Table 4). Surprisingly, no German texts were identified by this process. But the later work from the second French bestseller list is included on the English side of this new list (Table 2). So are the two translations of *Equilibration of cognitive structures*. (Although this effect goes away when citations for both versions are added together, so this entry in the table could perhaps be struck-out.) And one book is entirely new to us.

**Table 4.** Piaget’s most highly “under-rated” texts (by calculated score), presented in limited form using standard deviations as a shortcut for determining significance.

English vs. French	German vs. French
*The child’s conception of space* (−1)**	[none reach the level of 2*SD*]
*Biology and knowledge* (−0.91)**	
*The equilibration of cognitive structures* (−0.85)**	

This new book, *The Child’s Conception of Space* (Piaget & Inhelder, 1948/1956) can best be understood as part of Piaget’s experimental response to Kant: the categories of experience cannot be given a priori if they can be shown to develop. For this to have been received so underwhelmingly by English-speaking audiences then reinforces Piaget’s impression—as he put it in commenting on Flavell’s (1963) introductory textbook—that the view presented of his work in English was more psychological than epistemological, whereas his own perspective was the reverse (see Piaget, 1963, pp. viii–ix).

From the perspective of psychologists, this book on space presented *yet still more results* from the Genevan psychological factory: replications of things already known or suspected from earlier works, albeit sometimes using interesting new methods to demonstrate them. From an epistemological perspective, however, the book was quite significant: it connected Piaget’s psychological research with current problems in the philosophy of science and delivered on the promise of his inaugural speech when he accepted the Chair in Philosophy of Science and Psychology at Neuchâtel (Piaget, 1925). It also did so experimentally, rather than through theoretical or philosophical argumentation (e.g., Piaget, 1949). And it directly foreshadowed the explicit mission of Piaget’s genetic epistemology as a scientific research program (see Burman, 2021).

Of course, it is not necessary to know all of this to enquire further. Simply look at the dates: *The Child’s Conception of Space* was the direct sequel to *Psychology of Intelligence*, and—in French—it also appeared alongside another book on how children construct their conception of geometry (Piaget et al., 1948/1960). Now that we know where to look, we then also see that these were followed by volumes in Piaget’s in-house epistemology series that were also dedicated specifically to this topic (Bang et al., 1964; Bang & Lunzer, 1965). But these associated texts are otherwise easily missed: Piaget was not a named as a co-author on the latter, and instead edited the series in which they were published. (Indeed, the Swiss foundation that administers the rights to translate texts published under his aegis does not list it among his works.)

In French, however, *The Child’s Conception of Space* is included on the Top 10 list. (It also only just narrowly missed the cutoff for inclusion in Table 1 as a bestseller.) But in English, it ranks in the middle of the middle tier. It is, in other words, perceived as having been of middling importance: something to be passed over, in translation, without a problem. And I think this is indeed because it was understood by English audiences as psychology, rather than as experimental epistemology using psychological methods.

### Controlling for Language While Using Citation Density

**Figure 5.**
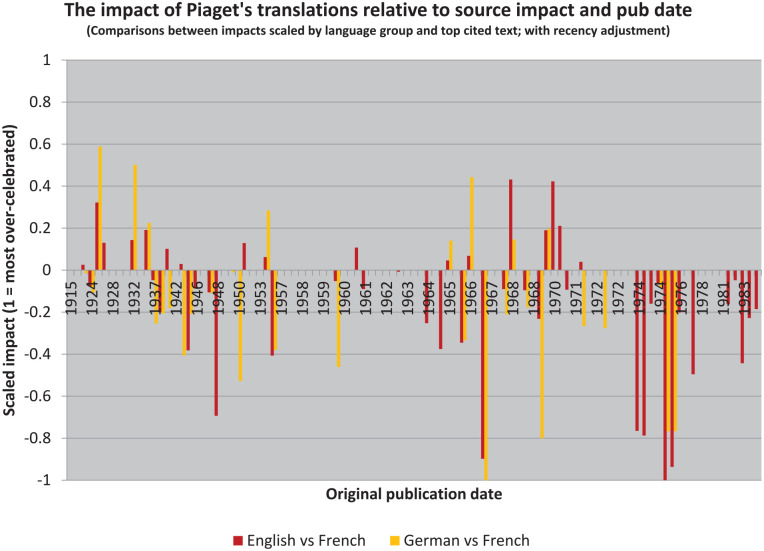
Controlling for the number of years in which texts could accummulate citations, then also controlling for the number of citations per year in the source language, highlights certain texts as needing further investigation.

Similar results can be derived from these data after taking citation density into account (Figure 5). Yet, curiously, no book is “over-rated” from this perspective (following our reliance on the short-cut of two standard deviations). It seems that taking historicity into account eliminates this category from both the English and the German texts. We see, however, that many more books are “under-rated” in translation. And that there are many more of these more recently.

The main difference in using citation density, as compared with raw counts, is that the Matthew Effect is at least partially controlled-for. As a result, more recent books are highlighted (Table 5). Thus, for example, *Biology and Knowledge* (Piaget, 1967/1971) is identified as a highly “under-rated” text in both English and German, whereas *Success and Understanding* (Piaget, 1974/1978) is listed only for English. (The two translations of *Equilibration of cognitive structures* were identified as well, but this effect again disappears when their counts are combined.)

**Table 5.** Piaget’s most highly “under-rated” texts (by density-weighted score).

English vs. French	German vs. French
*The equilibration of cognitive structures*, 1975/1985 (−0.94)**	*Biologie und Erkenntnis*, 1967/1974 (−1.0)** [Biology and knowledge]
*Biology and knowledge*, 1967/1971 (−0.90)**	
*Success and understanding*, 1974/1978 (−0.79)**	

Although it was not my explicit intent to use quantitative tools to identify Piaget’s new theory, we do indeed find signposts here pointing to it: *The Equilibration of Cognitive Structures* is the theoretical summary of psychological experiments presented in *Success and Understanding* (and other books from that era), whereas *Biology and Knowledge* represented a return to and update of the original biological meta-theory that informed Piaget’s earliest works. Of course, we still need to understand what we are being shown to recognize the significance. But this is also what we expect from the Wundt Argument (and the associated identification of the omission of the *Völkerpsychologie*). These data do not *make* such arguments on their own; they do not speak for themselves. Rather, they suggest possible investigations. (Indeed, that is what is valuable about such methods: they offer a beginning, not an end [see Burman, 2018a].)

## Toward a Generalized Argument

It seems reasonable to conclude, on the basis of this quick quantitative review, that Piaget was affected by the same sort of importation process as was Wundt (shown previously and conclusively by slower qualitative investigations). I therefore also propose, following that earlier example, that changes were made in the meaning of Piaget’s research program while it was being re-interpreted for new audiences: certain aspects were stressed, de-emphasized, or left out.

That said, however, my goal is not really to talk about Piaget. Instead, I want to *use “Piaget”* to speak in a new way about this process of meaning-change. The goal now is therefore to demonstrate that this general form is needed, and to do that by removing as much of the time-related variance as possible from a simple temporal model of the texts’ impact such that what remains can be attributed unambiguously to differences between the contexts (which can then be investigated). Note, too, that this relies on translations having been made; when they were not, that itself should be treated as reflective of *a choice* driven by context and interests (which can also be investigated).

### Raw Citation Data

Beginning at the simplest possible place, we will start with the basic figures. The mean publication date and standard deviations for the books examined are as follows: 1959.8 (16.3) in French, 1969 (16.5) in English, and 1973.4 (7.4) in German. Given that Piaget was born in 1896, and died in 1980, the very late average in even the original French is surprising: for most authors, “retirement age” is not the *midpoint* of a career. Yet beyond this, and noting the average delays in translation of roughly ten and fifteen years, there is very little of substance to say about publication dates alone. (Except perhaps to quote Quine’s impression that the Later Piaget was “publishing fast and furiously” to justify his grants—qtd. in Burman, 2021.)

The earliest translations into English all fall more than two standard deviations from the mean on the low end. This supports the accepted belief that Piaget was “discovered” by the Americans early on and then “rediscovered” later: first as a sociologist, then later—paradoxically—as a psychologist who ignored the social (Burman, 2015). Indeed, the early translations fall far outside the expected range for a continuous sequence and were followed by a decades-long gap. What is more interesting, however, is that Piaget also dismissed those earliest works up to and even including *Moral Judgment*. We thus find a mirror of Quine’s objection, regarding Piaget’s publishing, but at the opposite end. As Piaget later reflected on those early works: “I published them without taking sufficient precautions concerning the presentation of my conclusions, thinking they would be little read and would serve me mainly as documentation for a later synthesis” (Piaget, 1952, p. 246). That this matches up almost exactly with our findings is coincidental, of course, because the choice of two standard deviations is conventional. But it is certainly made more interesting by our having identified *The Child’s Conception of the World* (Piaget, 1926/1929) as having been the most highly overrated book celebrated by English audiences as representing Piaget’s thought (Table 3).

On the high end of the distribution, all of the books published in English and French fit within two standard deviations of the mean but for one exception: the new edited volume on “reason” (Henriques et al., 2004). This project was started in the last year of Piaget’s life and had been assumed abandoned. That belief was clearly incorrect. Unfortunately, however, as with many of Piaget’s later works, this book has not yet been translated (but for the introduction, see Piaget, 2004/2006). And as with the Bang book from the EEG series, this was also published without Piaget’s name in the byline. So most readers do not recognize it for what it is.

In addition to the posthumously completed book, at least two other unpublished books have also been discovered in the papers from Piaget’s home (see Ratcliff & Burman, 2015). These fill in details omitted from the abridgement that became *Understanding Causality* (Piaget & Garcia, 1971/1974; cited as “to appear” in Piaget & Garcia, 1983/1989, p. 283). For obvious reasons, however, these were not included here either: there are no translations, and indeed no originals to which those might have been compared.

The German case is somewhat more complex. Two books in our sample sit beyond two standard deviations from the mean on the distal side: *Das moralische Urteil beim Kinde* in 1954 (known in English as *Moral Judgment*; Piaget, 1932/1932), and *Die Bildung des Zeitbegriffs beim Kinde* in 1955 (*The Child’s Conception of Time*; Piaget, 1946/1969). The first is an obvious choice for an early translation: it is one of Piaget’s best-known books in both English and French. (And highly overrated in its German reception; Table 3.) But the second seems like a peculiar choice: it is considered a lesser work in English and of slightly better than middling importance in French. Yet it is also tied to the neo-Kantian program mentioned earlier, and—given the American preference for applicable practical insights and discomfort with grand theories—that is perhaps why it was considered “more relevant” to a German audience than to the Americans.

Two German translations also sit above two standard deviations on the proximal side: *Die Entwicklung des inneren Bildes beim Kind*, translated in 1990 (*Mental imagery in the child*; Piaget & Inhelder, 1966/1971), and *Intelligenz und Affektivität in der Entwicklung des Kindes*, translated in 1995 (*Intelligence and Affectivity*; Piaget, 1954/1981). Neither is especially well-known in English.

The first is a study of spatial and geometrical reasoning and can thus be understood as an update of *The Child’s Conception of Space* (Piaget & Inhelder, 1948/1956) and synthesis with Bang’s results reported in Piaget’s epistemological series (Bang et al., 1964; Bang & Lunzer, 1965). The second is an extended treatment of lecture notes from a course at the Sorbonne in 1953–1954, in which Piaget discussed the “energetics” and “structuring” of reason. This is of course in conflict with the “cognitive” interpretation that American audiences apply in interpreting Piaget, which is interesting, but the reason for the German publisher’s choice to translate this volume over another is not clear to me.

### Periodization of Raw Citation Data

**Figure 6.**
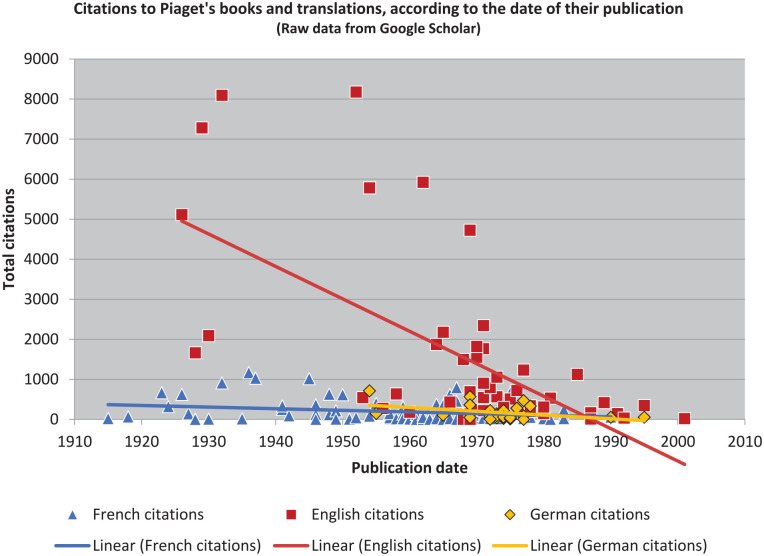
Older books are cited more often in all three languages considered. This then sets up the Levi Effect: what an author means, afterward, is a reflection both of what they have said and of how the earliest works to be translated were received. Works not translated effectively do not exist: there are no equivalents, and these sources therefore cannot serve as constraints on interpretation.

We can use the raw citation data to get a sense of the relationship between time of publication and impact in each language. This then highlights certain features of Piaget’s different publishing careers, such as the long gap prior to the “rediscovery” in English and the large cluster of translations in both English and German in the late 1960s through the late 1970s (Figure 6). But it is the regressions that provide us with a better tool in thinking through our sought-after elimination of temporal effects.

An astonishing 42% of the variance in the English raw citation counts can be accounted for simply by the date of publication (*r* = −.65). The German figure is lower, but still substantial: 16% (*r* = −.40). Compare these figures with the French data: only 6% of the variance in citations can be accounted for by date of publication (*r* = −.25). This difference is also suggestive: although there does indeed seem to be a Matthew Effect at work, it is more extreme in translation (the Levi Effect).

Why might this be? Piaget chose the order of presentation of his ideas in French. But in translation, this choice was influenced by other interests. We can then see a strong order-effect at work in influencing the interpretation of works published afterward. Our goal is then to try to eliminate this effect by reasonable arithmetical transformation. The remaining variance can then be attributed to errors and—more importantly—contextual differences: things normally investigated by traditional historical methods.

### Periodization of Citation Density

**Figure 7.**
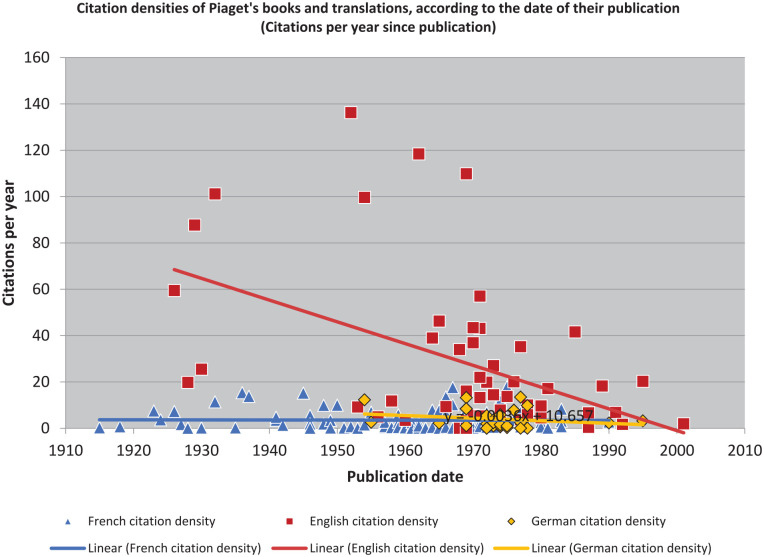
Switching to a consideration of the number of citations per year in which it has been possible to cite a text (“citation density”) shows much the same thing: older texts are typically cited more often. They frame the interpretation of later texts, and skew the resulting understanding.

The shift from raw citation counts to citation density has an immediate and readily apparent consequence, especially in English (Figure 7). The strength of the relationship—as illustrated by the slope of the regression line, where slope is derived by solving for *m* in *y = mx + b*—is much reduced (from *m* = −81 to *m* = −0.94).^22^ And the modeled variance is reduced to 23% (*r* = −.48). In German, the change is proportionally similar (from *m* = −9.3 to *m* = −0.11). And variance attributable to order effects is reduced to 4% (*r* = −.21).

It is with the French data, however, that our goal is properly achieved. The slope is nearly flat (from *m* = −4.0 to m ≈ 0). And the variance is also reduced practically to 0 (*r* = −.04). In other words, when citation density is taken into account, there is no detectable relationship between the impact of a text in its source language and its original date of publication. The order effect is thus removed. (*Perceived importance* is then due to other factors, including what the text actually says.) From this, we can therefore proceed with greater confidence to reexamine the earlier language-controlled analyses as well.

### Periodization of Raw Citations, Controlling for Language

**Figure 8.**
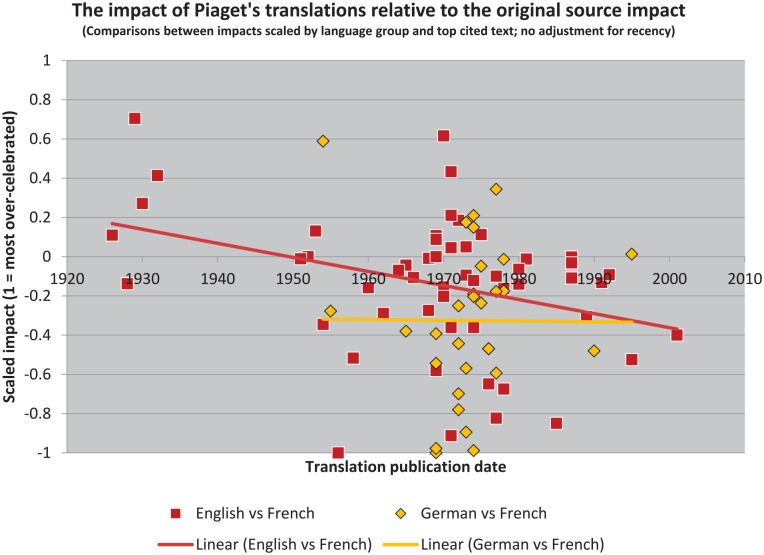
The understanding of Piaget in English is more skewed than the understanding of Piaget in German, when controlling for the impact of the same texts in French.

By controlling for both language and date of publication, using our over-under calculations, we can eliminate differences in the importance of the underlying text when treated referentially. The focus is then entirely on how the different audiences perceived their *variora*: the senses they had. And the results are again dramatic (Figure 8).

In English, the slope of the regression is made flatter still (*m* = −0.01). And the variance is reduced to 14% (*r* = −.38). In German, too, the slope is almost perfectly flat (*m* ≈ 0), with variance effectively eliminated as well (*r* ≈ 0).

The clustering of the bulk of German translations in the 1970s could be masking an effect by constraining them to a single era: the interpretation of these texts was not interrupted by a rupture that would have allowed meanings to drift during the intervening years. But the English texts still show a small order effect.

### Periodization of Citation Density, Controlling for Language

**Figure 9.**
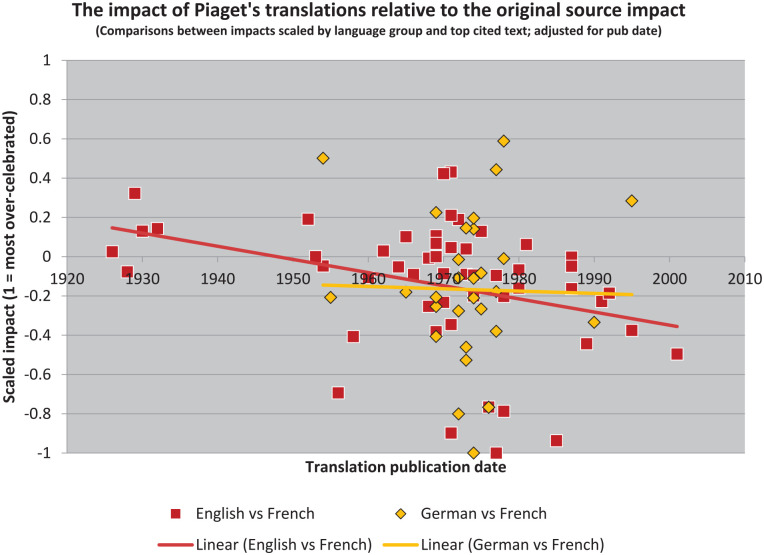
The skew persists in English even after removing all sources of skew in French. The Levi Effect is therefore shown conclusively to be a problem: when considering works in translation, whether linguistically or historically, one must take care not to interpret them through the mistaken mirror.

Curiously, the same analysis using citation density shows no appreciable change: the slopes are effectively the same, and the variance is only minimally different (from 14.1% to 13.7% for English; Figure 9). From the perspective of trying to control for impact per year, this suggests that the relative impact per year is therefore skewed consistently.

Eliminating the first five English translations as possible outliers (Piaget’s juvenilia) also has only minimal effect: the slope again remains effectively unchanged (from *m* = −0.007 to *m* = −0.009). And variance again drops only minimally: from 13.7% (*r* = −.37) to 8.7% (*r* = −.29). In other words, it seems safe to conclude that the English data show a small effect that is nevertheless sufficient to be of interest in its own right. (This not the case in German, *r* = −.02, where the question is more direct: *by what criteria were the texts themselves chosen to be translated?*)

This all suggests that there is more to Piaget, especially in English, than readers assume given what is available: his work was translated in several periods, separated by ruptures and different interests, which have gone largely uninvestigated. But the way to do that, as has been demonstrated with Wundt, is *history*. This is the means by which we can come to see things that are not always consistent with present perceptions, and yet which are nevertheless no less real.

## Conclusion

My purpose in doing this work has not been to suggest that Piaget’s status ought to be elevated to be on a par with Wundt’s. (This would be celebratory—reverential—and therefore forbidden by current professional standards in history.) Instead, my goal has been to make it easier to articulate *the argument*: earlier historians showed that our understanding of “Wundt” changed as a result of the process of importation, into American Psychology, and consequent meaning-change. And now we have a method that can suggest this occurred with others too, as we look to identify new opportunities for histories that can more quickly suggest to us what might have previously been missed (following Burman, 2018a).

Piaget has been convenient in making this argument because he wrote so many books over such a long period, and so many of these were translated. Indeed, he wrote so much that we can extend the methods to take a further step and compare the language-controlled impacts against each other. This then identifies only one book that is overrated by the English relative to its impact in both French and German: *Science of Education and the Psychology of the Child* (Piaget, 1930–1965/1970). And indeed, the identified-text is exemplary of exactly the way Piaget criticized what has turned out to be the most influential public understanding of his work: how to speed up cognitive development using educational interventions (see the discussion of “the American question” in Hall, 1970, p. 31). This shift in focus toward education reform, especially after Sputnik, also highlights for us that *meaning* is not innate to these objects that move: that book uses Piaget’s words, but they do not have his sense. Nor do many others whose meaning nevertheless seems “obvious” (see Burman, 2021).

In other words, I am proposing that translation itself represents a kind of boundary-work—between scientific cultures separated by language—and furthermore that it occurs as a result of the negotiations described by Gieryn (1995) through which epistemic spaces get filled with “science” (i.e. trustworthy knowledge defined according to the standards of the extant cognitive authority). Examining one therefore affords the means to see the other. I am not content, however, to consider the individual translations as “boundary objects” in his sense. I instead want to use them as leverage, collectively, so we can see something bigger that could not otherwise be seen. Indeed, this is what everything here has ultimately been leading up to.

### The Indigenization Argument

From all of this, I want to present a more general version of the Wundt Argument that I will call the “The Indigenization Argument.” That then recognizes the traditional (qualitative) historical view and also its augmentation here with a new (quantitative) approach, thereby broadening the kinds of evidence we can consider in investigating meaning-change as science moves. The goal is furthermore not to limit this to Piaget or even to psychology: comparisons of different receptions could easily be replicated for other authors with a large number of books that were translated and then imported into new contexts.

The simplest way to clarify this generalized version of the argument is to refer to one of the originators of the original argument. Thus, as Kurt Danziger (2006) explained,

. . . the process now described as “indigenization” is one that has been a feature of modern psychology from its earliest days. What became the universalistic science of psychology had its roots in distinctly local traditions of science and philosophy in nineteenth-century Europe. British evolutionary biology, French psychiatry, and German experimental physiology each gave rise to different ways of conceptualizing and investigating human subjectivity scientifically (Danziger, 1990), and the export of each of these forms was always accompanied by considerable modification of the original. This process had some elements in common with what is now referred to as indigenization, but when one turns from the circulation of psychological knowledge within Europe to the export of this knowledge to the United States one encounters indigenization on a massive scale. (pp. 215–216)

He continued,

It is only to be expected that when a science is transplanted from one part of the world to another there will be some shift of priorities, some change in the topics that receive the most attention, some adaptive modification of the techniques considered most appropriate. (p. 217)

And indeed it is these shifts, changes, and modifications that I have sought to detect, so those differences can be investigated (see also, for example, Carson, 2007, 2014).

One way that this line of thinking has developed is to argue that *all psychologies* are indigenous psychologies, including the British and French and German exports that came to inform what is now primarily an American export (e.g., Marsella, 2013; Teo, 2013b). Some of what we observe is then a function of external, nonintellectual factors that constrained what could be produced in those different contexts (e.g., Pickren, 2007; Pickren & Rutherford, 2010; Walsh et al., 2014). And this realization opens up consideration for people and ideas that have traditionally been ignored or neglected for reasons that, after investigation, seem strange (Burman, 2015; Burman et al., 2015; see also, for example, Guthrie, 1976; Held, 2020; Pickren, 2009a, 2009b; Rutherford, 2015; Winston, 1998). However, in keeping with my goal of going broad, I also want to highlight another line of thinking here: a parallel interest arising from within D’Andrade’s (1995/2012) cognitive anthropology and its goal of advancing an “analysis of meaning” (pp. 16–30).

From that perspective, what I have presented seems consistent with a macro take on the classic analyses of “similarity judgments” (pp. 48–54). Except that I have inverted this following the historian’s preference for identifying and investigating differences. Still, I have relied on the responses to translations to interrogate those differences at the level of the collective, with the goal of identifying differences between “semantic networks” (pp. 62–64) and to use mathematical tools to investigate their “salient features” (pp. 64–66). This in turn highlights potential conflicts in the “folk taxonomies” (pp. 92–100) operating underneath each indigenous psychology. And these provide the frames through which external meanings are imported and made local, thereby informing which thoughts or actions make sense in the moment to perform (the different effects of which we can observe, as I have done in considering translations as *variora*).^23^

Psychologists will probably be familiar with this kind of interaction primarily through the influence of Ian Hacking’s (1986, 1995, 1998, 2007) discussions of the Looping Effect. But by this comparison, we are also led to a new perspective of name-attribution in the “anthropology of science” (Atran, 1993) and also to a reconsideration of the importance of historical and disciplinary taxonomies—or, more specifically, the ontological categories to which these names refer^24^—in grounding both scientific and experiential worldviews (Hacking, 1993; Sankey, 1998; also Cassin et al., 2004/2014; Lomas, 2018).

Briefly, then, my more general version of the argument is this: when meanings move, different audiences will interpret them in different ways that are consistent with the extant meaning-systems operating as *forms* that precede and then structure *contents*. In other words, imports are received into a framework that preexists them and which filters out the unthinkable bits by judging them to be irrelevant rather than equivalent. Investigators can thus use the identified differences to learn something new about those audiences and their receiving contexts. And indeed, this can be understood as a regularization of Barrett’s Law, in considering the public understanding of science: “*not everyone who might read the productions of scholarly writers is an expert in the fields discussed*” (Burman, 2012b, p. 99; emphasis as in the original). In articulating our specialist research findings, we must therefore also concern ourselves with different “publics” (Pettit & Young, 2017). Because the words we use do not speak for themselves (pace Dennett, 2017).

### Why Is This Not Agnotology?^25^

What I have presented could easily be misunderstood as a demonstration of *the ignorance* of audiences in relation to the full meaning of imported source-words, and particularly of English and German readers and translators in understanding the meaning of French concepts. And thus the preceding could be misinterpreted as “agnotology” (see Proctor & Schiebinger, 2008; also Kourany & Carrier, 2020).

This is not implausible. Ignorance is sometimes mentioned as one of the causes of the American misunderstanding of Wundt: his American students spoke in English, which he understood but could not himself speak, and he answered in German (Blumenthal, 1977, pp. 17–18). This seems like it might have been sufficient. Yet it is also said that he supported the passing of a law banning all foreign students from German universities as a result of their poor mastery of the language (by Tawney in “In Memory of Wilhelm Wundt by his American Students,” 1921, p. 179). And we see similar problems in the translations of Piaget, where one particularly noteworthy example is the rendering of *orage* as “orange” instead of “thunderstorm” (noted by Smith, 2009, p. 30).^26^

Ignorance and incompetence, however, are not my preferred interpretations for the effects shown. The shift is too consistent, and too easily connected to the order of publication. In addition, it seems to me that adopting an agnotological approach conflicts with the norms of the disciplines involved: just as Piaget sought to understand the justifications given in support of his children’s knowledge-claims, Kuhn argued that the historian of science must work to understand how improbable or unthinkable propositions were justifiable in the context of their time.^27^ (Teo, 2013a, distinguishes between historicist and philosophical approaches to agnotology, and only the former would be compatible with what I intend.)

The so-called ignorance that I have operationalized here as *variance in reception*—by the receiving target audience (in English or German) relative to the originating source (in French)—must be interpreted historiographically as arising from differences in equivalence, between the source and the targets, that in turn altered the audiences’ interpretations of what the translated texts meant. From this perspective, differences in impact are the result of the receiving audience (including the translator) having brought a different reading to the texts than was understood in the source language. And that, in turn, must be investigated further; typically, by producing histories of the differences that altered the meanings of the received texts.

This observation must also itself be examined further, however, in considering what it is that our linguistic (historical) signposts are pointing to. Indeed, one could reasonably advance the argument that overcoming ignorance is exactly the issue: there is a *correct interpretation*, represented by the French originals, and all that needs to be done is to make a comparison to evaluate the truth-status of any claims made in translation. We can even imagine that, in the future, such a task might be performed automatically by artificial intelligence (AI).

This is then the realm of extensional definitions, in Carnap’s (1947/1956) update of Frege (1892/1980), with the French originals serving as the basis for evaluating truth claims comparatively. From this perspective, too, Jevons’ and the Early Wittgenstein’s truth-tables can serve as prototypes: a given observation is compared with the set of known truths, not only in the French originals but also generally (if we imagine a universal encyclopedia),^28^ and that observation is then determinable by reference to be true or false. In translation, however, this is not straightforward.Hence Quine’s (1960) indeterminacy of translation: different language groups could be said to refer to different translation manuals (using different operations) in evaluating the meanings of propositions, when comparing descriptions and the objects described, and so it may not be possible—formally, fully, universally—to evaluate claims between partially conflicting systems (i.e. to do so consistently and completely).

From this philosophical perspective, we cannot rely on even sophisticated AIs to say definitively what the proper meaning of “Wundt” or “Piaget” is *in translation*. Nor can we make automated judgments about whether their texts have been *correctly* rendered, because we cannot apply the truth-table of one system to the meaningful propositions of another. An AI (or a philosopher) might therefore declare victory, in the face of this indeterminacy, and—absent any evidence of errors or inconsistencies—say simply that there are different equally valid language games being played by different equally valid language groups (following the Late Wittgenstein).^29^ More grist for the historian’s mill, in other words. There is, however, a less relativistic alternative that derives from Kuhn’s further development of his historicist approach to incommensurability.

The meaning-loss that occurs in translation, when making comparisons between two systems having nonoverlapping parts, need not simply be accepted. Although in *Structure* he appealed to translation, Kuhn’s (1993/2000) eventual response was to advocate for language-*learning*: “I was wrong to speak of translation” (p. 238). Yet this learning also need not be interpreted dialectically to imply the synthesizing of a new truth-table that subsumes the relevant aspects of both languages and can then be referred to extensionally (with Quine, 2001/2008). Instead, Kuhn advocated for an intensional (with-an-ess) approach to truth evaluation.

As he explained, he was more interested in his later works in understanding differences in meaning-in-context than in making comparisons to an explicit and incommensurable standard:

What is it that translation must preserve? Not merely reference, as I have argued, for reference-preserving translations may be incoherent, impossible to understand while the terms they employ are taken in their usual sense. That description of the difficulty suggests an obvious solution: translations must preserve not only reference but also sense or intension. (Kuhn, 1983/2000, p. 50)

And earlier in the same essay,

The translator’s choice of particular English word or phrase . . . is ipso facto the choice of some aspects of the intension of the French term at the expense of others. Simultaneously it introduces intensional associations characteristic of English but foreign to the work being translated. (Kuhn, 1983/2000, p. 48)

This is then what I propose that my quantitative analyses have shown: there are different intensions between language groups, and these in turn altered the perceived significance of the *variora* considered. Equally valid though these interpretations may have been philosophically, as the texts were received into the extant scientific language-games (barring any errors), they were also demonstrably *different* in particular ways.^30^ And the historian seeks to enquire as to why and how, as well as what the participants were blinded-to as a result.

We are thus led by this to a further insight that I would like to offer as a result of the generalized Indigenization Argument, following Kuhn’s observations about what it is that History can contribute to Science. In short: contemporary historiography ought not only to be referential, but it also ought to be sensible. And by this I mean that it should be sensitive not only to the interests of the past but also to the interests of the receiving audience. Because these shape how the words used will be understood; how they derive their *sense*.

From a set of equally valid interpretive possibilities, specialist historians provide evidence, contextualize, and explain—and indeed present *arguments*—regarding what they consider to be a justified interpretation in the present of the past. And then when new evidence is discovered, new arguments can suggest new interpretations. But it is not *the past* that changes; it is *our understanding* in the present that changes. Revision thus becomes *re-vision*; representation, *re-presentation*.

We do not have access to the past as it was. We only have access to the evidence that has been preserved. We therefore also cannot appeal to an objective and timeless source against which to evaluate historical truth claims. There is not only no truth-table, but there also *can be* no truth-table. Instead, what historical evidence can be said to mean always derives from an evaluation relative to what we know: new observations are considered in relation to other evidence, the meanings of which can be considered as reflecting a shared web of belief (Quine) or arising from the implications deriving from a lexical network of senses and references (Kuhn after his postscript to *Structure*).

The result is still relativistic, but only if we adopt a static perspective. And that is not historical. So we could also rephrase, in recognition of the found-order effects, and replace “relative” with “generatively entrenched” (after Wimsatt, 2007). Thus, the result of an Early Kuhnian revolution—which is to say the replacement of one meaning-full system of entrenchment with another—is reconceived, with the Later Kuhn, as “working in a new world” (Hacking, 1993). The meaning of “socially constructed” then becomes *socially constrained* (Hacking, 1999). Indeed, my generalized argument could also itself be renamed “the domestication argument” (separate from Danziger but still connected to him and his role in the discourse). And maybe it should be.

In short: the present is biased by our assumptions about the world that we are working in, and the sources that shape our perception of it. Our apparently-unproblematic inheritances, and what we *consider* relevant or equivalent, constitute the mistaken mirror that produces the illusions to which scientists are blind and which historians find so problematic. But using the methods that I have shown, we can now at least detect the amount and direction of the skew. Then we can investigate, and—especially—listen to those who could not previously be heard.^31^

From this perspective, *truth is not dead* (contrary to what many post-Early Kuhnian antirealist relativists have argued). It merely needs to be reconceptualized as being part of a developmental process (i.e. with apologies, “de-agnotologically”).^32^ Alternative interpretations are thus constrained by the extant evidence to afford a set of equally acceptable possibilities that are all indeterminately true *without further investigation*. To understand this in greater depth, formally, we can then appeal—as Kuhn (1989/2000) did, and Piaget (1981/1987, 1983/1987) did too—to the modals of Possible Worlds Semantics.

To wit: given all of the evidence that we presently have of the past, what *might* have been the case at that time and what *must* have been the case? What in the imagined plurality of past worlds (to which we don’t have direct access) was *possible*, what was *necessary*, and what was *impossible*? And by what criteria have these judgments been made? Or, in other words: I think psychological scientists who are concerned with replication would benefit from asking how historians know what they know of events or periods that they could never even conceivably have visited or witnessed. And, indeed, how the resulting evidentiary perspective is sometimes also more trustworthy than the descriptions of those who did. After all, memory can be a mistaken mirror too: recollection, without sense, is not replication.

## Notes

1. This was popularized in *Leviathan and the Air-Pump*: “virtual witnessing involves the production in a *reader’s* mind of such an image of an experimental scene as obviates the necessity for either direct witness or replication” (Shapin & Schaffer, 1985, p. 60). They note, also, that this is active; not passive.

2. Indeed, Quine (1996/2008) later wrote: “I am thus won over to Thomas Kuhn and others who have insisted that observation is inseparable from theory” (p. 477). Yet this remains problematic in its details: his “observation sentences” that comprise science are still driven *by observations*. Hence the relevance, here, of the mistaken mirror: the focus of science should not *solely* be on what we can see, because this could be illusory. However, indeterminacy then also led Quine to much the same conclusion as Kuhn: “The finality of an experiment is historical, not logical” (p. 477).

3. Citing J. G. Miller as the source, Quine used the memorable phrase “ontology recapitulates philology” (in the epigram to his 1960; as well as in two unpublished essays, written a half-century apart, collected by Føllesdal & Quine, 2008, pp. 146, 182). For his approach, this can be understood to mean that a scientific worldview (what is real) is a function of the ways in which observation sentences might be coherently constructed (how things can be described). Here, I show that there is an additional intervening layer that needs to be understood and theorized. And I propose that this reflects socially inherited norms and values that define *what’s relevant and good* (see Burman, 2013, 2020a). As a counterpart to *paradeigma*, we must therefore also consider *aisthanomai*: the governing aesthetics (Burman, 2012a, 2014).

4. My apologies for using J. S. Mill’s “connotations” here when I should perhaps have stuck with Frege’s “senses.” But it seemed clearer, in explaining, because English readers only have access to Frege in translation. The issue, briefly, seems to me to be one of connotation via meaning-reference versus denotation via object-reference.

5. The distinction between *reverential history* and *referential history*—to distinguish between celebratory and critical historiography—was recently made by Burman (2020a). Here, that gains some additional formal power: while both *refer*, reverential history also includes senses imparted by the author’s celebratory attitude.

6. Much the same thing could be said of, for example, Freud in Cambridge (see Forrester & Cameron, 2017).

7. We also see this in Watson’s (1913) Behaviorist manifesto: he appealed to Darwin (pp. 161–162), and contrasted with Titchener (p. 164). Of course, when one actually looks, there were more “Americans” in Leipzig than just Titchener (see Benjamin et al., 1992).

8. The typical response of textbook authors has been to point to Piaget’s “genetic epistemology” as the higher-order counterpart to his developmental psychology of intelligence, or of cognition, but without explaining what this was. (For an archive-driven history that is meant to remedy this lack, see Burman, 2021.) The new theory, however, is something else entirely: “epigenetic epistemology” (see, for example, Burman, 2013, 2016, 2019, 2020a).

9. The allusion here is to “the neglect of the foreign invisible” (Burman, 2015). That can be considered the qualitative precursor to what here becomes a general quantitative argument.

10. These two sources report on the same study. Indeed, the first is an abridgement of the second. Because the relevant portions are nearly identical, I proceed with the source that is now easier to find.

11. Following Gieryn (1995), the form is prior to the content. The content then gets filled in—“performed”—after the form has been negotiated. So to understand the content, we first have to understand the form (which historians usually refer to as “context” but which I mean more formally).

12. As a result of the available tools, the kind of scholarship that is done today is also quite different (cf. Benjafield, 2017; Green & Feinerer, 2015, 2016; Young & Green, 2013).

13. Other databases could certainly have been used. Because the focus is on relative differences, the differences between what each databases reports is more a function of their coverage than of the named-reference sought. Here, therefore, I have used the database with the broadest reach.

14. Books and pamphlets are included according to the categorization produced by the Fondation Jean Piaget in Switzerland: http://www.fondationjeanpiaget.ch/fjp/site/bibliographie/index.php. The best source I know for titles in all three languages is Richard Kohler’s online bibliography: http://www.richardkohler.ch/piagetbiblio.htm

15. I am grateful to Maarten Derksen for pushing me to emphasize this point.

16. These different rounds of data collection were undertaken to inform several talks with different emphases, including at Cheiron (2014), the Jean Piaget Society (2014), and the Swiss Psychological Society (2015). The resulting discussion helped to inform several of the studies published in between, from which this revision also benefited.

17. Porter’s (1995) “trust in numbers” thus becomes “trust in databases” (Burman, 2020c).

18. Piaget (1972) noted this in his address at the first meeting of the Jean Piaget Society (see also Furth, 1972).

19. In historical investigations of the intellectual context informing these sources, I have referred to related ideas as level-spanning downward completion (Burman, 2013), meaning projection (Burman, 2016) and morphisms across levels in a spiral—the comparison between which in turn powers abstractions and generalizations (Burman, 2020b).

20. This first manipulation was easily done in an Excel spreadsheet using the large function: with book title, publication date, and raw citation count in the first three columns, a new column is calculated by dividing the relevant cell in each row (the individual book’s citation count) by the largest number from the “raw” column (the book with the highest citation count in that language). Once defined, the formula can then be populated across the rows. Of course, Excel will automatically increment the cell references during this process. And although this is convenient for updating the reference to the “raw” column, it introduces an error inside the function. As a result, the function needs to be corrected for all rows. With this done, the corrected column can then be copied and pasted into another column (or to another sheet) for application with another language group.

21. To construct the illustration, I first removed from consideration those works for which there is no translation in both target languages. Then I constructed a new formula, which required several columns in Excel. I subtracted the scaled language-controlled source (French) impact from the scaled language-controlled target impact (separately in English and German), and then—to preserve the scaling—I used the abs function and the large function to divide by the highest-density text in the target language. This enabled the division to use the absolute value of the product of the subtraction, which then ensured that the results remained within the expected range for a scalar comparison (±1).

22. Benjafield (2017) explains that a slope of −1 is distinctive of “pink noise,” which he describes as characterizing phenomena that follow Zipf’s Law (p. 512). My goal, in the manipulations that follow, is therefore to reduce this to “white noise” (a slope of 0). The difference and why it matters, very briefly, is akin to that between complexity and randomness: one can be productively investigated for underlying causes, and the other cannot (see also Benjafield, 2016, pp. 135–136). (I am grateful to Andrey Feuerverger for his efforts in ensuring that I understood the difference.)

23. Related research using other methods has been reported by Burman et al. (2015), Green et al. (2015a, 2015b), and Burman (2018b, 2018c).

24. This flips Quine’s recapitulationism (see Note 4) in the same way as Baldwin, Piaget, and Waddington flipped Haeckel’s (see Burman, 2019).

25. I rewrote the conclusion to add this section upon learning of the death of Les Smith (1943–2020). We had several versions of this conversation over the years, and I always benefited from his perspective. I wish I could have heard his thoughts on this latest iteration.

26. A child asks their father, “How can you tell the difference between the start of one *orage* and the end of another?” (paraphrasing). Depending on what *orage* refers to, whether “orange” or “thunderstorm,” this is either a very astute question—by a precocious thinker—or a highly problematic one that is perhaps in fact indicative of special needs.

27. The apparent similarity is no accident. Kuhn attributed it to Piaget (see Burman, 2020a).

28. This seems straightforward philosophically. Looked at historically, however, the appeal overlooks the work done by the encyclopedists in constructing *the categories* into which knowledge fits: the meaning-full containers that preceded the observations of objects that could be placed inside them (see especially Darnton, 1984/2009).

29. I am grateful to Hank Stam for pointing this out. Like so many good suggestions, it was obvious after he said it. And yet I was blind to it until he did.

30. In my favorite nonfiction book of all time, *Le Ton Beau de Marot*, Hofstadter (1997) examines this philosophically rather than historically.

31. One might therefore be tempted to examine the relative rates at which the works of women are translated into different languages, for example, or those by members of different “racial” groups (where the meaning of this term is now redefined with reference to “racism” rather than to “race”).

32. The allusion here to deontological ethics is intended, except that what I have in mind is developmental and without need for references to a *codified* set of rules against which actions can be judged (following Piaget, 1932/1932; discussed in Burman, 2020a). The formal framework through which this then makes sense derives from the formalization of levels introduced to Piaget by Ladrière following the publication of Gödel’s *Dialectica* interpretation (Burman, 2016, 2020b). The results, however, were both explicitly evolutionary and developmental (Burman, 2013, 2019). As indeed we expect from Kuhn’s final unfinished book (see Burman, 2007; Hoyningen-Huene, 2015; Kuukkanen, 2013). I am grateful to Steve Fuller for encouraging me to stop waiting for this to be published, and to be more proactive in presenting my own version.
